# Expanding the spectrum of reactive arthritis (ReA): classic ReA and infection-related arthritis including poststreptococcal ReA, Poncet’s disease, and iBCG-induced ReA

**DOI:** 10.1007/s00296-021-04879-3

**Published:** 2021-05-01

**Authors:** Yoshinori Taniguchi, Hirofumi Nishikawa, Takeshi Yoshida, Yoshio Terada, Kurisu Tada, Naoto Tamura, Shigeto Kobayashi

**Affiliations:** 1 Department of Endocrinology, Metabolism, Nephrology and Rheumatology, Kochi Medical School Hospital, Nankoku, 783-8505 Japan; 2 Department of Internal Medicine, Chikamori Hospital, Kochi, Japan; 3 Department of Internal Medicine and Rheumatology, Juntendo University School of Medicine, Tokyo, Japan; 4 Department of Internal Medicine and Rheumatology, Juntendo University Koshigaya Hospital, Saitama, Japan

**Keywords:** Reactive arthritis, Infection-related arthritis, Postinfectious arthritis, Spondyloarthritis

## Abstract

Reactive arthritis (ReA) is a form of sterile arthritis that occurs secondary to an extra-articular infection in genetically predisposed individuals. The extra-articular infection is typically an infection of the gastrointestinal tract or genitourinary tract. Infection-related arthritis is a sterile arthritis associated with streptococcal tonsillitis, extra-articular tuberculosis, or intravesical instillation of bacillus Calmette–Guérin (iBCG) therapy for bladder cancer. These infection-related arthritis diagnoses are often grouped with ReA based on the pathogenic mechanism. However, the unique characteristics of these entities may be masked by a group classification. Therefore, we reviewed the clinical characteristics of classic ReA, poststreptococcal ReA, Poncet’s disease, and iBCG-induced ReA. Considering the diversity in triggering microbes, infection sites, and frequency of HLA-B27, these are different disorders. However, the clinical symptoms and intracellular parasitism pathogenic mechanism among classic ReA and infection-related arthritis entities are similar. Therefore, poststreptococcal ReA, Poncet’s disease, and iBCG-induced ReA could be included in the expanding spectrum of ReA, especially based on the pathogenic mechanism.

## Introduction

The development of sterile arthritis after bacterial infection of the gastrointestinal or genitourinary tract is called reactive arthritis (ReA). Several bacteria are known to induce ReA, including such as *Shigella* (excluding *S. flexneri, S. sonnei*), *Salmonella typhimurium, S. enteritidis, Campylobacter jejuni, Yersinia enterocolitica, Y. pseudotuberculosis,* and *Chlamydia trachomatis* (Table 1).

**Table 1:** Pathogens of ReA

Definite causes of classic ReA
Gastrointestinal pathogens
*Salmonella* species
*Campylobacter jejuni* and *Campylobacter coli*
*Yersinia enterocolitica* and *Yersinia pseudotuberculosis*
*Shigella flexneri*; less commonly, *Shigella sonnei* or *Shigella dysenteriae*
*Clostridioides difficile*
Genitourinary pathogens
*Chlamydia trachomatis*
*Mycoplasma* species
Respiratory pathogen
*Chlamydophila pneumoniae*

Probable and possible causes of ReA
Bacille Calmette-Guérin (intravesicular), *Ureaplasma urealyticum*, *Bacillus cereus*, *Clostridium difficile*, *Escherichia coli*, *Helicobacter pylori*,
*Lactobacillus, Neisseria meningitis serogroup B, Pseudomonas, Streptococcus* species

In 1969, Ahvonen et al. proposed that reactive arthritis (ReA) be defined as aseptic or nonsuppurative arthritis following microbial infection of sites other than joints [1]. However, the term ReA is often loosely and widely used to refer to the occurrence of sterile nonsuppurative arthritis after microbial infection. Arthritis following hemolytic streptococcal tonsillitis or upper respiratory tract infection (i.e., poststreptococcal ReA [PSRA]) originally reported in 1982, aseptic ReA accompanying tuberculosis (Poncet’s disease), and ReA after intravesical instillation of bacillus Calmette–Guerin (iBCG) therapy are also considered ReA as they develop after the onset of another infection, and this has often caused confusion in the definition of ReA [2–4].

Considering these circumstances, at the 1999 International Workshop on Reactive Arthritis, it was proposed that the definition of ReA be restricted to HLA-B27-related arthritis associated with spondyloarthritis (SpA) symptoms and limited to arthritis involving organisms that cause urogenital, intestinal, or, in part, respiratory tract infections. In addition, it was proposed that, with the exception of septic arthritis, any other post-infectious nonsuppurative arthritis be referred to as infection-related arthritis [5, 6]. Therefore, post-infectious arthritis is fundamentally categorized as ReA (classic ReA, Reiter`s syndrome), infection-related arthritis, and post-infectious viral arthritis [7]. Of the post-infectious viral arthritides, ReA associated with human immunodeficiency virus (HIV) infection is most often reported and discussed in the literature. However, it was concluded that ReA is related to other infections that patients with HIV are exposed to, not to HIV infection itself [8, 9].

Despite the abovementioned facts, the term ReA is commonly used in a wide sense from the viewpoint of pathogenic mechanism. The purpose of this article is to review the epidemiology, clinical characteristics, treatment, prognosis, and differences between classic ReA, PSRA, Poncet’s disease, and ReA following iBCG therapy and clarify whether these entities are considered separate or one spectrum.

### Search strategy

We searched PubMed/MEDLINE and Japan Medical Abstracts Society databases for articles published between January 1958 and February 2021. We searched for articles and abstracts published in English or Japanese using the following keywords: “reactive arthritis,” “postinfectious arthritis,” “infection-related arthritis,” “poststreptococcal reactive arthritis,” “streptococcus-related arthritis,” “streptococcus” + “reactive arthritis,” “tonsillitis” + “arthritis,” “tonsillitis reactive arthritis,” “Poncet’s disease,” “tuberculosis arthritis,” “tuberculosis reactive arthritis,” and “BCG” + “arthritis OR reactive arthritis.”

All authors screened and reviewed titles and abstracts to identify studies examining ReA, classic ReA, and infection-related arthritis, including poststreptococcal ReA, Poncet’s disease, and iBCG-induced ReA. The articles were independently reviewed, and relevant information was extracted. A total of 64 articles were selected and included in this narrative review. We referred to the principles of writing a narrative review when designing our search methodology [10].

### Epidemiology

1. Classic ReA
  In a study in Norway, where the prevalence of HLA-B27 is high, the incidence of ReA after chlamydial infection or enterocolitis was reported to be 4–5 per 100,000 persons, which is similar to the reports of the Finland study [11, 12]. In addition, a survey of patients infected with *Shigella*, *Salmonella*, *Campylobacter,* or *Yersinia* by questionnaire that was conducted using questionnaires and laboratory tests showed that 7–12% of patients developed ReA [13–16], and other studies reported similar results [17].

On the one hand, according to a systematic review published in 2016, 3–8% of patients with *Chlamydia trachomatis* infection developed sexual intercourse-related ReA [18]. Furthermore, it was reported in a study conducted in Japan that, of 123 patients with chlamydial infection, only 1 patient (0.8%) developed ReA [19]. Interestingly, in a study of 8145 participants in India, there were no cases of ReA, and the prevalence of ReA was 0% [20]. Thus, geographic factors influence the pathogenesis, incidence, and prevalence of ReA. 

The sex ratio for post-enteric ReA is nearly 1:1; however, venereally acquired ReA is more often diagnosed in men (Table 2).

**Table 2:** Comparison between classic ReA, PSRA, Poncet’s disease, and iBCG-ReA

	Classic ReA	PSRA	Poncet’s disease	iBCG-ReA
Triggers	*Chlamydia,* *Campylobacter,* *Salmonella,* *Shigella, Yersinia, *etc	*Streptococcus*	*Mycobacteria*	*Bacillus Calmette-Guerin*
Pathogen dynamics	Intracellular parasitism	Extracellular	Intracellular parasitism	Intracellular parasitism
Number of joints affected	Oligoarthritis	Polyarthritis	Oligoarthritis	Polyarthritis
Sex ratio	1:1 (post-enteric) Male predominant (venereal)	1:1	1:1	1:1
HLA-B27 (%)	50–80	< 10	20–30	10–40
Axial involvement	Yes	Rare	No	No
Extra-articular manifestations	Yes	Not frequent	No	Yes
Tendency to chronicity	Yes	No	No	No
Improvement with antibiotics	No (Yes in Chlamydia)	Yes	Yes	No

*ReA* reactive arthritis, *PSRA* poststreptococcal reactive arthritis, *iBCG-ReA* intravesical Bacillus Calmette-Guerin therapy induced reactive arthritis, *HLA* human leukocyte antigen

(2) PSRA
  The reported annual incidence of PSRA in the United States is 1–2 per 100,000 persons [21, 22]. In a retrospective survey of pediatric patients admitted between 2000 and 2015 to 323 hospitals all over Japan, there were 44 patients with acute rheumatic fever (ARF) and only 21 cases of PSRA [23]. The sex ratio of patients with PSRA is 1:1.

(3) Poncet’s disease
  Musculoskeletal symptoms are reported in 10–19% of patients with tuberculosis, but Poncet’s disease, also known as aseptic ReA, is rare [24, 25].

(4) ReA following iBCG therapy for bladder cancer

iBCG, also known as *Mycobacterium bovis*, administration is a long-standing treatment for bladder cancer. Although it has not been conclusively determined that iBCG therapy has an antitumor mode of action, the potential mechanism can be roughly divided into three categories: (i) innate immune response, (ii) adaptive immune response, and (iii) direct action [26]. ReA is known to be an adverse reaction or complication of iBCG therapy. The sex ratio of patients with ReA following iBCG therapy is 1:1. In Western countries, the incidence of ReA following iBCG therapy is approximately 0.5–1% [27, 28]. Our survey showed that the incidence in Japan is approximately 2% [29]. Due to the high tuberculosis infection rate in Japan, BCG vaccination before the age of 1 year is now established and its administration is enforced as a law in Japan. Although the reasons for the high incidence of ReA following iBCG therapy in Japan compared to that in Western countries remain unknown, patients who previously received BCG vaccination may develop a booster effect from the next round of iBCG therapy.

### Etiology and pathogenesis

The role of HLA-B27 as a genetic factor in the pathogenesis of ReA has garnered considerable attention because a study reported that 50–80% of patients with ReA have HLA-B27 [30]. Although the prevalence of HLA-B27 in patients with ReA varies, in the general population, the incidence of ReA increases with an increase in the frequency of HLA-B27. Moreover, compared with ReA patients without HLA-B27, patients with HLA-B27 are more likely to experience acute and severe disease courses, extra-articular symptoms, and symptom chronicity [31].

A recent hypothesis on the pathogenesis of ReA with a focus on the role of microbiological factors is presented below [32]. In ReA after chlamydial infection, the urinary tract is the site of bacterial invasion, causing persistent intracellular infection of monocytes, and in ReA after intestinal infection, bacteria persist in the submucosal layer of the intestine. Similar to the transfer of chlamydia-infected monocytes to joints, viable organisms or bacterial products of intestinal infection may also transfer from the intestinal wall to joints via blood circulation. *Chlamydiae* are metabolically active (expressed mRNA) and can persist as chronic infection of synovial fibroblasts, whereas enteric pathogens such as *Salmonella* rapidly degrade within synovial fibroblasts, resulting in synovial fibroblasts with intracellular “bacterial ghosts” or microbial products. Yersinia antigens were demonstrated within phagocytes from the joints and blood of patients with ReA up to 17 years after the first documented infection [33]. They can become a source of antigens in joints and cause T-lymphocyte activation. Subsequent changes in the balance of Th2/Th1–Th17 cytokines affect the acute arthritis, resolution of arthritis, and chronicity of arthritis [32, 34].

Alternatively, the pharynx, tonsil, and bladder are sites of bacterial invasion, which causes activation of local innate immune cells such as natural killer cells in PSRA and ReA following iBCG therapy. The activated innate immune cells may then transfer to several joints, resulting in the development of arthritis. Moreover, in ReA following iBCG therapy, similar to classic ReA, bacterial DNA may also reach the synovial cavity either directly during bacteremia or by transport within lymphoid cells or monocytes [34].

*Salmonella sp*., *Yersinia sp*., and *Shigella sp*. are classified as facultative intracellular bacteria that are capable of living and reproducing either inside or outside host cells, and *Chlamydia* and *Mycobacterium* are classified as obligate intracellular bacteria that cannot live outside host cells (Table 2).

In contrast, *Streptococcus pyogenes* is classified as an extracellular bacteria as it resides outside host cells. *Streptococcus sp*., *Staphylococcus sp*., *Pseudomonas sp*., and other extracellular bacteria, some of which are able to produce biofilm patches around themselves, are capable of escaping contact with antibiotics, phagocytosis, and/or autophagy inside a macrophage or monocyte. Recent studies have shown that these extracellular bacteria survive inside CD14^+^ cells in tonsils [35]. Tonsils have been investigated on the basis of pathogen reservoir theory considering it was revealed that infection of tonsils occurs at multiple foci, that the microbiome is polymicrobial, and that bacteria are present throughout the tissue [36]. Therefore, the persistence of inviable *Streptococcus* and other extracellular bacteria in the CD14^+^ cells of tonsils leads to transfer to joints and induction of chronic or recurrent inflammation known as aseptic arthritis.

In our small study on PSRA, HLA-B39(16) was significantly demonstrated in patients with PRSA [37], and HLA-B39(16) is reported to have an amino acid sequence that is closely resembles that of HLA-B27 [37]. We have never observed HLA-B27 in any patients with PSRA who we have examined.

### Clinical symptoms

**Fig. 1:**
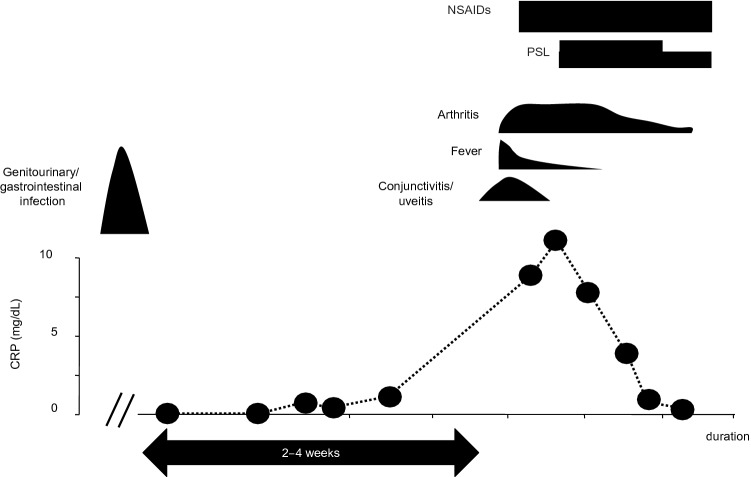
Typical clinical course of classic ReA. Typically, 2–4 weeks after the antecedent infection, which is usually a gastrointestinal or genitourinary tract infection, patients develop asymmetric oligoarthritis, conjunctivitis or uveitis, and urethritis. However, some patients might require several treatments for severe or prolonged symptoms, and the treatments could shorten the natural duration of the disease

1. Classic ReA
  ReA is predominantly an oligoarthritis that can also have spinal involvement. ReA usually has an acute onset. Typically, 2–4 weeks after the antecedent infection, patients develop asymmetric oligoarthritis, conjunctivitis or uveitis, and urethritis (Fig. 1). In approximately half the patients with ReA, all symptoms resolve within 6 months, and, in most patients, symptoms disappear within 1 year [6, 38]. However, some patients might require several treatments for severe or prolonged symptoms (Fig. 1), and the treatments could shorten the natural duration of the disease.

### Antecedent infection

Urogenital and intestinal infections that cause ReA are characterized by urethritis and diarrhea. A preceding infection may be clinically asymptomatic or identified only by testing [11]. The main microorganisms that cause the genitourinary, gastrointestinal, and respiratory tract infections that are precedent infections of ReA as proposed in the 1999 International Workshop on Reactive Arthritis are shown in Table 1 [5, 6, 32, 39].

### Musculoskeletal signs and symptoms

Lower-limb monoarthritis or oligoarthritis often occurs as a typical peripheral lesion. Arthritis persists for several weeks to 6 months but is usually transient. However, it may recur in approximately 15–50% of cases. Enthesitis may also follow antecedent infection, and suspicion of ReA increases with the presence of pain, swelling, or heat sensation at the enthesis site of the Achilles tendon, plantar fascia, quadriceps tendon, or patellar tendon. Dactylitis indicating sausage digits also occurs in approximately 40% of patients [32, 39, 40]. Although axial lesions occur less frequently than peripheral lesions, sacroiliitis is observed in approximately 20% of patients. Spondylitis (especially the rare spondylitis of the lumbar vertebrae) is often observed in patients with HLA-B27 (Table 2) [32, 39, 40].

### Extra-articular symptoms

Extra-articular manifestations are not uncommon. During the course of ReA, aseptic urethritis, and cervicitis, especially secondary to chlamydial infection, occur in approximately 60% of patients. In men, mild dysuria, mucopurulent urethral discharge, prostatitis, epididymitis, and balanitis circinata may occur. Dysuria, vaginal discharge, purulent cervicitis, and vaginitis may occur concurrently in women [32, 39, 41]. The eyes are affected in 50–70% of patients, and unilateral or bilateral conjunctivitis is often observed. Episcleritis, anterior uveitis, and keratitis are also observed with variety. The prognosis of eye lesions is sometimes poor. Therefore, if severe redness, pain, and photophobia are observed, an ophthalmologic examination should be promptly recommended [32, 39, 41]. In terms of mucocutaneous lesions, oral ulcers and keratosis blennorrhagica are found in approximately 10% of patients, but erythema nodosum is rarely found. Cardiac lesions such as aortitis, aortic regurgitation, heart block, and pericarditis are observed in less than 10% of patients [32, 39, 42].

**Fig. 2:**
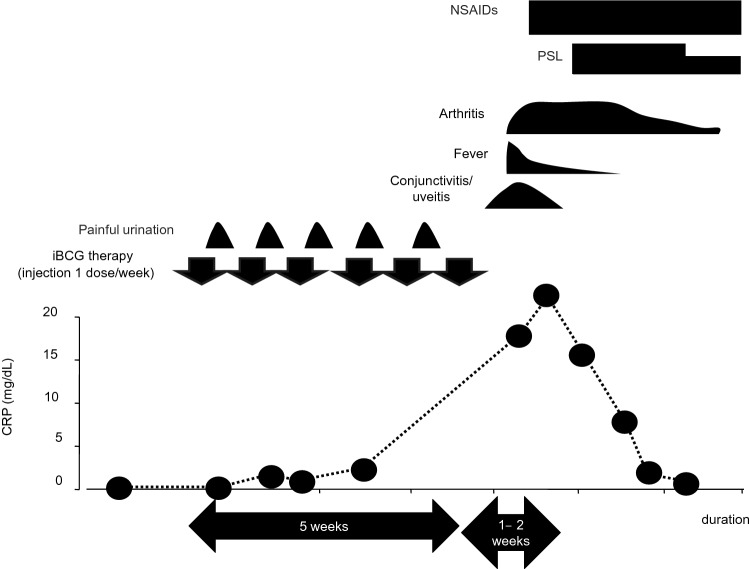
Typical clinical course of ReA following iBCG therapy. Painful urination first occurs between the 4th and 6th round of iBCG therapy, and arthritis and conjunctivitis (or uveitis) are observed 1–2 weeks after the last round of iBCG therapy. This timeline suggests that ReA follows iBCG therapy

(2) PSRA
  The basic joint symptoms of PSRA are usually similar to those of classic ReA. The age distribution of PRSA appears bimodal, with a peak incidence at ages 8–14 and a secondary peak at ages 21–37 [43]. Clinically, PSRA causes acute asymmetrical non-migratory polyarthritis. If the patients have recurrent tonsillitis, arthritis may also be recurrent and last from 2 weeks to 10 years [43, 44]. None of the patients examined had residual joint damage or deformity. Axial skeleton involvement, such as sacroiliitis, is uncommon in patients with PSRA (Table 2) [45, 46]. Arthritis of the sternoclavicular joints was noted in our patients [44]. Out of 21 patients, 2 had uveitis, 3 had annular erythema, and 3 had non-specific abdominal pain [44]. Annular erythema and non-specific abdominal pain are also clinical manifestations of ARF. We first reported two cases of uveitis in PSRA patients [47]. It is noteworthy that uveitis and arthritis were induced in Lewis rats after systemic injection of streptococcal cell walls, and streptococcal antigens were found in the eyes and joints of the experimental animals [48]. Carditis was not observed in our adult patients as reported in the literature [43].
  The duration of arthritis in adult patients with PSRA before diagnosis as mentioned in our previous report is 29.8 ± 35.9 months on average, with a range of 0.5–120 months and a median of 24 months. Padhan et al. reported that the mean duration of arthritis in adult patients was 29.25 ± 18.51 weeks [49]. Thus, the duration of arthritis in our patients before diagnosis was extremely long. Furthermore, the duration of arthritis before diagnosis was longer in patients who had undergone tonsillectomy than in patients who had not undergone tonsillectomy. It was noted that adult PSRA patients with chronic and/or recurrent tonsillitis were often misdiagnosed and/or inappropriately treated before they sought treatment at our clinic.

(3) Poncet’s disease
  Patients with active tuberculosis develop aseptic ReA with almost the same symptoms as classical ReA. Patients with Poncet’s disease typically exhibit non-erosive arthritis with extra-articular tuberculous lesions, often on the lungs and skin; these items have been proposed as diagnostic criteria. However, in these patients, sacroiliitis is mostly non-complicated, and this is characteristic of Poncet’s disease (Table 2) [24].

(4) ReA following iBCG therapy for bladder cancer
  Typical onset and symptoms are as follows: painful urination is first experienced between rounds 4 and 6 of iBCG therapy, and arthritis and conjunctivitis (or uveitis) occur 1–2 weeks after the last round of iBCG therapy before the onset of ReA (Fig. 2) [50]. Arthritis can involve the hands, knees, and ankle joints, and 50% of patients have polyarthritis, 40% have oligoarthritis, and 10% have monoarthritis. Arthritis occurs predominantly in the lower extremities. Furthermore, oligoarthritis has an asymmetric distribution. Enthesitis and dactylitis also occur in these patients. Concomitant occurrence of conjunctivitis and arthritis are observed in approximately 40% of patients. Conjunctivitis precedes arthritis in approximately 30% of patients, conjunctivitis is followed by arthritis in approximately 10% of patients, and the time of onset is unknown in approximately 20% of patients (Table 2) [27, 28, 50].

### Laboratory tests and imaging

#### Blood and joint fluid tests

Patients often present with arthralgia and joint swelling similar to rheumatoid arthritis (RA), but rheumatoid factor and antinuclear antibody are usually negative. The acute phase of ReA is characterized by an increased inflammatory response (increased C-reactive protein [CRP] level and erythrocyte sedimentation rate). HLA-B27 is found in 50–80% of patients with classic ReA, and positive findings are helpful. The frequency of HLA-B27 in patients with PSRA is not different to that in the normal population (Table 2). Poncet’s disease and ReA following iBCG therapy are less strongly associated with HLA-B27 than classic ReA (Table 2).

Confirmation of antecedent infection is the most important task. If ReA secondary to genitourinary infection is suspected based particularly on assessment of early morning urine, then culture and polymerase chain reaction test of urethral and vaginal secretions for chlamydia as well as measurement of serum antibody titer should be performed. If ReA secondary to enteritis is suspected, stool culture for *Shigella sp*., *Salmonella sp*., *Campylobacter sp*., and *Yersinia sp*. should be performed [32, 39]. Since some patients with HIV infection develop ReA before the onset of acquired immune deficiency syndrome, HIV antibody test should be performed.

**Fig. 3:**
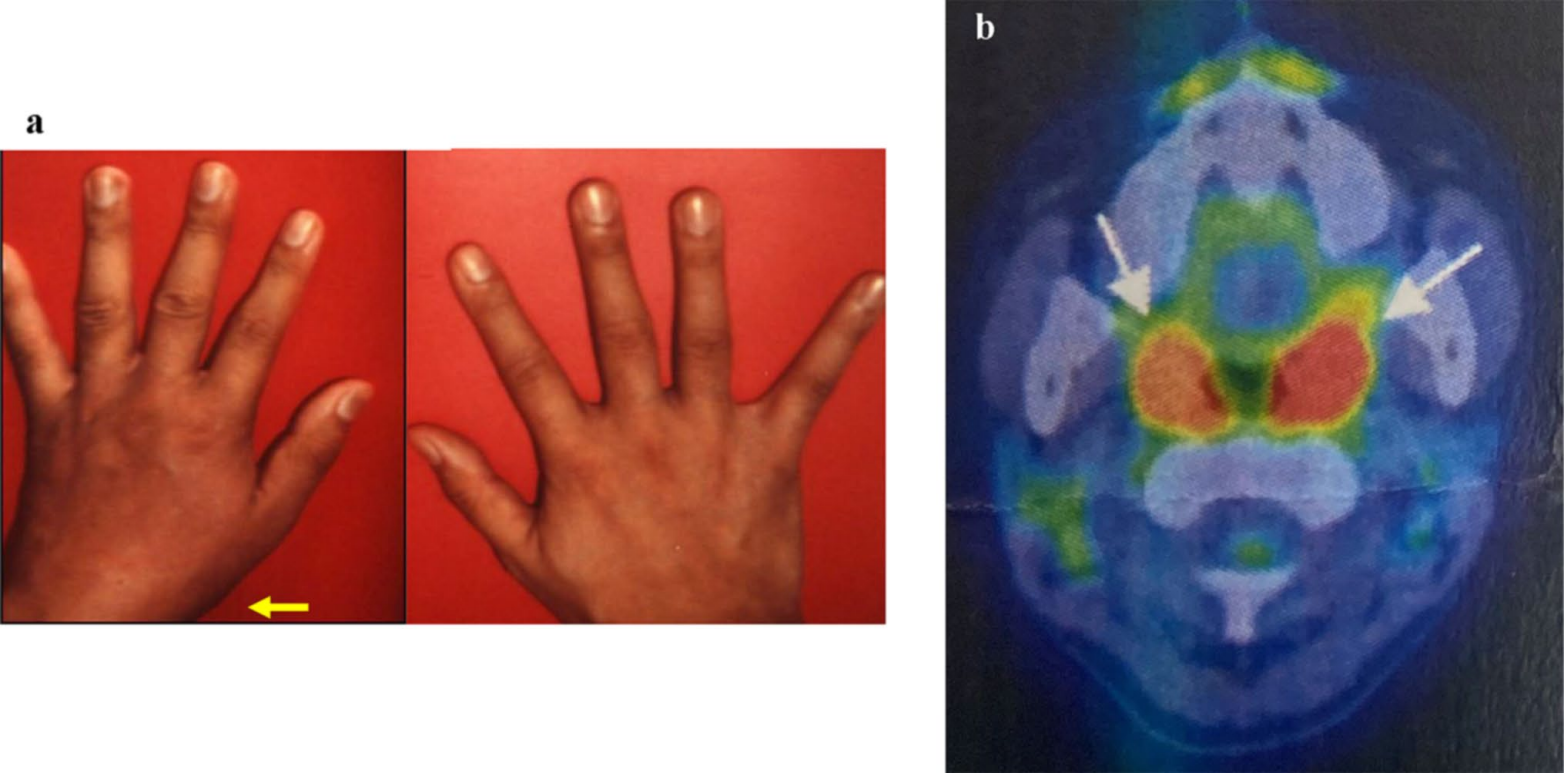
Arthritis of left wrist and image of patients with tonsillitis-related arthritis. Arthritis of either side of the wrist and tonsillitis recurred for 10 years. The left wrist inflammation improved 1 week after antibiotic therapy. *Pseudomonas aeruginosa* was the only bacteria found in a culture of the resected tonsils (a) [52]. Positron emission tomography/computed tomography shows the inflamed palatine tonsils of a 38-year-old woman with recurrent tonsillitis and arthritis (b)

If PSRA is suspected, antistreptokinase (ASK) antibody or antistreptolysin O (ASO) antibody titer should be measured. It is also important to be aware of the presence of tonsillitis and to consult an otolaryngologist. Regarding tonsillar stimulation test, tonsillar massage or manipulation results in increase in CRP level, white blood cell count, and body temperature as well as exacerbation of arthritis within 24 h [21, 44]. In our series of adult patients, which included patients with chronic and recurrent manifestations, 62% of patients were positive for ASO and/or ASK, and throat culture for group A streptococcus was positive in 57% of patients [44]. Further, reports indicate that up to 40% of patients are unable to demonstrate a history of streptococcal infection [51]. It was reported that since different bacterial groups are found throughout the tissue, a surface swab taken for culture will not provide a complete assessment of pathogenic organisms [36]. Hence, streptococcus and other bacterial species may be involved in this type of infection-related arthritis, especially in patients with chronic and recurrent manifestations. We previously encountered a 30-year-old man who had repeated episodes of tonsillitis and arthritis of either wrist that started when he was 20 years of age (Fig. 3a). Serological examinations for *Streptococcus* were not positive. Each time a throat swab culture was performed, *Pseudomonas aeruginosa* was found, and this bacteria was finally demonstrated in microabscesses on resected palatine tonsils [52]. Therefore, our diagnosis was ReA induced by tonsillar *Pseudomonas aeruginosa* infection or tonsillar *Pseudomonas aeruginosa* infection-related arthritis, but not PSRA.

In our study, anticardiolipin IgG antibody was positive in 8 of 13 patients with PSRA and 6 of 7 patients with infective endocarditis. This finding can be explained by the rich streptococcal cell wall component found in cardiolipin. However, increased anticardiolipin titers and no sign of thrombosis were noted in adult patients with PSRA during the observation period [53].

Synovial fluid analysis following aspiration revealed 10,000–50,000 white blood cells per high power field, with neutrophil predominance. The presence of bacterial components or DNA in the joint fluid of patients with ReA has been reported, but bacterial cultures were negative as no viable bacteria were present [32, 39, 44].

### Imaging

X-ray findings of peripheral arthritis and enthesitis include soft-tissue swellings or bony proliferations at the entheses. In patients with chronic sacroiliitis, erosion and sclerosis of the sacroiliac joint may be observed, and this finding is often unilateral. X-ray of spondylitis, unlike that of ankylosing spondylitis, show bony proliferations that usually extend horizontally to the long axis of the spine, and they are often asymmetrical [32, 39]. Ultrasonography and magnetic resonance imaging (MRI) can excellently detect peripheral synovitis and enthesitis. MRI of the sacroiliac joint and spine can show bone marrow edema of the sacroiliac and spinal joints in patients with active ReA and axial lesions [32]. Computed tomography (CT) scan or positron emission tomography/CT scan will be helpful in the demonstration of intratonsillar abscesses for patients with PSRA (Fig. 3b) [54].

### Diagnosis and differential diagnosis

1. Classic ReA
  Clinical diagnosis is based on the pattern of findings described above and on the differentiation and exclusion of other diseases. There is no single definitive diagnostic test. Although certain criteria were proposed in 1996 [55], they have not been established as diagnostic criteria. The most important task is to identify the antecedent infection based on a thorough medical history. The clinical diagnosis should be made through a diagnostic approach after adequately excluding differential diseases and evaluating the clinical characteristics [39]. Several differential diseases can cause acute monoarthritis or oligoarthritis, and they include septic arthritis, disseminated gonococcal infection, viral enteritis-associated arthritis, Whipple disease, inflammatory bowel disease (Crohn’s disease and ulcerative colitis), Behcet’s disease, crystal-induced arthritis, Lyme disease, and sarcoidosis [32, 39]. Clinical diagnosis of ReA should be made after distinguishing and excluding differential diseases based on the pattern of signs, symptoms, and findings.

(2) PSRA
  The presentation of sudden-onset fever and severe joint pain may be suggestive of acute septic arthritis. For every new patient with arthritis, it is important to confirm acute arthritis of large joints and take history of tonsillitis symptoms. If tonsillitis symptoms are present, ReA associated with tonsillitis and/or pharyngitis should be considered. ASK/ASO antibody titers should be measured, but a negative test cannot rule out PSRA. It is often noted that the palatine tonsils are situated in a deep tonsillar fossa, and inflammatory signs are not often observed on the surface of the tonsils in chronic and recurrent cases as described in the literature [36]. As with classic ReA, a thorough differential diagnosis should first be performed, and the relevant differential diseases should then be ruled out to make a clinical diagnosis [21]. It is noted that in cases of chronic presentation in patients who previously had seronegative RA or undifferentiated arthritis, clinicians should consider PSRA and ask the patients if they had episodes of recurrent tonsillitis.

(3) Poncet’s disease
  It is important to demonstrate the development of aseptic arthritis during antecedent active tuberculosis. The diagnostic criteria proposed by Sharma et al. are helpful for clinical diagnosis [56]. In other words, non-erosive arthritis has to be confirmed, other causes ruled out, and extra-articular tuberculous lesions confirmed. If tuberculin test is positive and hypersensitivity phenomena such as erythema nodosum are observed, then the patient probably has Poncet’s disease. Finally, definitive diagnosis is made after confirmation of good response to antitubercular drugs.

(4) ReA following iBCG therapy for bladder cancer
  As with classic ReA, clinical diagnosis of ReA following iBCG therapy is based on the pattern of physical and imaging findings and the differentiation/exclusion of other diseases. Although septic arthritis due to disseminated BCG infection after iBCG therapy is exceedingly rare [57], it is always necessary to exclude direct BCG infection. As with classic ReA, there is no single definitive diagnostic test and no established diagnostic criteria for ReA following iBCG therapy for bladder cancer. It is of paramount importance to bear in mind that ReA is a possible adverse reaction to iBCG therapy and to sufficiently inquire about iBCG therapy after thorough evaluation of symptoms. After adequate identification and exclusion of differential diseases, clinical diagnosis can then be made following evaluation of clinical characteristics.

### Treatment and prognosis

For effective management of ReA, it is necessary to consider treatment options that target the antecedent infection and symptoms including arthritis.

### Treatment of antecedent infection

For ReA secondary to enterocolitis, there is essentially no evidence that antibiotic therapy for enterocolitis is effective [58]. In contrast, patients with chlamydial ReA may benefit from antibiotic therapy [59]. In chlamydial ReA, it is also important to treat sex partners with antibiotics to prevent the so-called ping-pong infection. In cases of PSRA, an antibacterial drug that targets streptococci should be administered in combination with the drugs for arthritis [43, 60]. For Poncet’s disease, the initial consideration is to treat the underlying tuberculosis with antituberculosis drugs [24]. In cases of ReA following iBCG therapy for bladder cancer, it is important to discontinue iBCG therapy [27, 28, 50].

### Treatment of acute arthritis

Pain management with non-steroidal anti-inflammatory drugs (NSAIDs) is the main initial treatment for acute arthritis [32, 39]. Considering that many patients are cured spontaneously, NSAID administration for 2 weeks may be sufficient. Steroids are administered systemically when patients do not respond to NSAIDs or intra-articular steroid injection or when patients have polyarthritis. Treatment is often started with prednisolone (PSL: 20 mg daily for mild cases and 40 mg daily for moderate to severe cases); subsequently, the dosage is promptly reduced. Patients with acute ReA who do not respond to NSAIDs or steroids are administered disease-modifying antirheumatic drugs (DMARDs) such as salazosulfapyridine (SASP) and methotrexate (MTX) [32, 39].

Acute arthritis due to PSRA often ceases with the administration of NSAIDs and antibiotics. If there is no improvement, tonsillectomy should be considered in collaboration with an otolaryngologist who fully understands the ramifications of tonsillectomy with regard to PSRA. Typically, there will be marked resolution of arthritis within 3 weeks after tonsillectomy [43, 44, 60]. In the patients with Poncet’s disease, arthritis resolves between 1 week and 4 months after the initiation of antitubercular drugs [21].

For ReA following iBCG therapy, iBCG therapy should first be discontinued and NSAIDs should then be administered as first-line treatment. For patients with serious symptoms or patients who are non-responsive to NSAIDs, oral steroids (PSL: 10–20 mg daily) should be administered. Isoniazid, an antitubercular drug, may be administered concomitantly in some cases, in consideration of post-iBCG therapy. SASP and MTX may be administered to patients refractory to NSAIDs and steroids and to patients with chronic disease. Basically, these treatments mostly induce clinical remission, but there are reports of cases progressing to chronic persistent arthritis. Therefore, treatment for at least 3 months is desirable [27, 28, 50].

### Treatment of chronic arthritis

DMARDs are basically used for the treatment of chronic arthritis. In fact, in a placebo-controlled prospective study of patients with chronic ReA, treatment with SASP resulted in significant improvement in symptoms compared to placebo (62% versus 47%, respectively) [61]. MTX has been used as a surrogate agent for NSAIDs, steroids, and SASP in refractory patients. There are no research data to support MTX administration in patients with ReA, but MTX may be used in ankylosing spondylitis associated with peripheral joint lesions, and this is based on in-use experience [32, 39]. TNF inhibitors are considered as treatment for patients with chronic ReA and enthesitis or dactylitis when NSAIDs are ineffective. Furthermore, TNF inhibitors are considered when response to maximum dose of SASP or MTX for 3–4 months is inadequate for chronic arthritis. The efficacy of TNF inhibitors is supported by limited data that are based on case reports or case series [62]. However, tocilizumab, an IL-6 inhibitor, was reported to be effective against ReA. Th17 cell count is increased in the joints of patients with ReA, and IL-6 and IL-1 are involved in the induction of Th17 cells, and this shows the mechanism of action and effect of tocilizumab [63].

For patients with chronic chlamydia-induced ReA, antibiotic combination therapy with doxycycline, rifampicin, and azithromycin for 6 months improved joint symptoms in 17 of 27 (63%) patients. In contrast, 3 of 15 (20%) patients improved with placebo. Moreover, remission was achieved in 22% of patients in the antibiotic combination group and in 0% of patients in the placebo group, suggesting that antibiotic combination treatment is efficacious in patients with chlamydial ReA [50].

Our patients with PSRA were treated with antibiotics and NSAIDs, and 8 of the 21 patients showed no improvement; therefore, they underwent tonsillectomy and had no subsequent recurrence of arthritis or tonsillitis. It is important to note that bacteria are present throughout the tonsillar tissue even in patients with no symptoms of infection at the time of tonsillectomy [36].

### Prognosis

Most patients with ReA achieve complete or near-complete remission within 6–12 months after treatment. Retreatment after relapse may be required in 25%–50% of cases. Additionally, approximately 15–20% of cases become chronic, requiring continuous treatment. Some of these chronic patients develop signs and symptoms of ankylosing spondylitis or inflammatory bowel disease. ReA in patients with HLA-B27 is more likely to progress to chronic ReA and shift to chronic SpA with radiographic changes [32, 39, 64]. There is usually no joint destruction in patients with PSRA, but a few cases of cardiac involvement in adult patients have been reported.

### Limitations

The review and analysis were based on a search using solely PubMed/MEDLINE and Japan Medical Abstracts Society databases. A limited set of keywords and articles were selected by authors, and, therefore, some relevant studies might have been missed.

## Conclusion

From the viewpoint of pathogenic mechanisms, including typical and atypical intracellular parasitism, classic ReA and infection-related arthritis, including poststreptococcal ReA, Poncet’s disease, and iBCG-induced ReA, could be included in the expanding spectrum of ReA. However, considering the diversity in triggering microbes, infection sites, and frequency of HLA-B27, these are different disorders with similar clinical symptoms. Therefore, this review has improved our understanding of the similarities and dissimilarities between ReA and infection-related arthritis.
